# Neuroprotective Properties of a Standardized Extract from *Myracrodruon urundeuva* Fr. All. (Aroeira-Do-Sertão), as Evaluated by a Parkinson's Disease Model in Rats

**DOI:** 10.1155/2014/519615

**Published:** 2014-06-25

**Authors:** Iana Calou, Mary Anne Bandeira, Wellida Aguiar-Galvão, Gilberto Cerqueira, Rafaelly Siqueira, Kelly Rose Neves, Gerly Anne Brito, Glauce Viana

**Affiliations:** ^1^Department of Physiology and Pharmacology, Faculty of Medicine, Federal University of Ceará, Rua Cel. Nunes de Melo 1127, 60430-270 Fortaleza, CE, Brazil; ^2^Faculty of Pharmacy, Dentistry and Nursing, Federal University of Ceará, Rua Alexandre Baraúna 949, 60430-160 Fortaleza, CE, Brazil; ^3^Faculty of Medicine Estácio of Juazeiro do Norte, Avenida Tenente Raimundo Rocha 515, 63048-080 Juazeiro do Norte, CE, Brazil

## Abstract

*Myracrodruon urundeuva* Fr. All. (*Anacardiaceae*) is a Brazilian medicinal species, which is common to the Northeastern Brazilian semiarid region, whose stem-bark is widely used in folk medicine. It is an endangered species, presenting as main bioactive components tannins and chalcones. In this work, we studied the neuroprotective effects of a standardized extract from cultivated* M. urundeuva* (SEMU), in a model of Parkinson's disease. Thus, a unilateral injection of 6-OHDA was done into the rat right stratum. The animals were submitted to stereotaxic surgery, then treated with SEMU (5, 10, 20, or 40 mg/kg, p.o.) for 2 weeks, subjected to behavioral tests, and euthanized for striata dissections and neurochemical, histological, and immunohistochemical analyses. We showed, for the first time, that SEMU reverted behavioral alterations seen in the 6-OHDA-lesioned group and partially blocked the decrease in DA and DOPAC contents. The numbers of viable neurons and TH immunopositive cells were increased by SEMU. In addition, the SEMU-treated 6-OHDA groups showed lower numbers of GFAP and OX-42 immunopositive cells. The neuroprotective action of SEMU is possibly related to the antioxidant and anti-inflammatory properties of* M. urundeuva*, pointing out to its potential use in the prevention or treatment of neurodegenerative conditions, such as Parkinson's disease.

## 1. Introduction

*Myracrodruon urundeuva* Fr. All., known as “aroeira-do-sertão”, is an arboreal medicinal species that belongs to the* Anacardiaceae* family, common to the “caatinga” (shrub vegetation) of the semiarid Northeast Brazil region. The stem bark of this species is popularly used for gynecological conditions, mainly as a* postpartum* medication, and this use is justified by the anti-inflammatory and analgesic properties of this plant, as previously demonstrated by us in several experimental models [1, 2]. Other studies [3] have pointed out the presence of tannins which are possibly responsible for the plant natural resistance to degradation. Besides tannins,* M. urundeuva* presents dimeric chalcones that also contribute to its pharmacological effects [4].

Tannins are defined as phenolic compounds of high molecular weight (ranging from 500 to more than 3000 Daltons), mainly present in the plant bark and wood. They bound to proteins, forming insoluble or soluble tannin-protein complexes. They have been closely associated with the plant defense mechanisms against mammalian herbivores and insects [5].

Natural antioxidants such as phenols, flavonoids, and tannins are increasingly attracting health sciences researches, because they are natural disease preventing, health promoting, and anti-aging substances. Antioxidants may serve the task of reducing oxidative damage in humans, induced by free radicals and reactive oxygen species under oxidative stress conditions. These conditions can cause DNA and protein damage, lipid peroxidation, cancer, ageing, and inflammatory activity. In the last decade, there has been an upsurge of interest in the therapeutic potentials of medicinal plants as antioxidants, in reducing free radical induced tissue injury [6].

Phenolic compounds are secondary metabolites of plants, with different activities such as protection against pathogens and predators, mechanical support, attraction of pollinating animals, and protection against ultraviolet radiation [7, 8]. These compounds constitute a chemically heterogeneous group, containing phenols (which present a functional hydroxyl group in an aromatic ring) in their basic structure. They differ structurally, from simple molecules such as phenolic acids to highly polymerized compounds, such as tannins, comprising different classes. However, the main phenols in the human diet are the phenolic acids, flavonoids, and tannins [9].

Considering the importance of* M. urundeuva* to the popular medicine in Northeast Brazil and its elevated risk of extinction, we decided not only to develop cultivation techniques but also to study, in a comparative way, the chemical and pharmacological properties of the native and cultivated plants, shown previously [10]. Now, the main focus was on neurodegenerative diseases, mainly because the great majority of studies with* M. urundeuva*, including ours, were on its anti-inflammatory, analgesic, and antimicrobial properties, but studies on its effects on the CNS are practically inexistent.

Thus, the objectives of the present paper were to study, for the first time, the behavioral and neurochemical effects of a standardized extract of* M. urundeuva*, prepared from the cultivated young plant, on a model of Parkinson's disease in rats. Besides, some histological and immunohistochemical assays were also carried out, in an attempt to elucidate the mechanisms of action of the bioactive plant components.

## 2. Material and Methods

### 2.1. Drugs and Reagents

Ketamine (5% Vetanarcol) and xylazine (2% Kensol) were purchased from König (Santana de Parnaiba, São Paulo, Brazil). The reagents for HPLC determinations (standard monoamines) were from Sigma-Aldrich, St Louis, MO, USA, while those for immunohistochemistry assays were from Santa Cruz, CA, USA, Chemicon, Temecula CA, USA or DAKO, Santa Barbara, CA, USA. All other reagents were of analytical grade.

### 2.2. Plant Material

The plant was collected near the city of Iguatu (Ceará state, Brazil) and identified by Professor Afranio Fernandes of the Department of Biology of the Federal University of Ceará (UFC). It is presently cultivated in the UFC Medicinal Plant Garden and a voucher specimen is deposited at the Prisco Bezerra Herbarium, under the number 14,999.

### 2.3. Preparation of the Fluid Extract from* M. urundeuva* (SEMU)

The fluid extracts were prepared from stem and leaves of young plants (around 70 days of development), which were dried in the oven at 40°C (with air circulation), according to the Brazilian Pharmacopeia (2010). Briefly, the dried material (30 g) was grinded and submitted to two extractions. The first one used a mixture of glycerol : ethanol : H_2_O (1 : 6 : 3, v/v) for maceration of the dried material, for 6 h in a percolator, resulting in 24 mL of the extracted liquid material. The residue was submitted to a second extraction with a mixture of ethanol : H_2_O (2 : 1, v/v), up to exhaustion. The resulting liquid was evaporated in a water bath at 60°C, until a syrup consistency. This syrup was added to the liquid material from the first extraction, the volume was completed to 30 mL with distilled water and the final mixture was filtered.

### 2.4. Determination of Total Polyphenol and Tannin Contents in the Fluid Extract of* M. urundeuva*

For the preparation of the standardized fluid extract stock-solution (SEMU), all steps, including extraction, dilution, and spectrophotometric readings, were carried out in the dark, using free-CO_2_ distilled water. Then, 1 g fluid extract (prepared as described above) was transferred to an Erlenmeyer, containing 150 mL distilled water. After boiling, the mixture was placed in a water bath, at 80–90°C for 30 min, followed by cooling in an ice bath, and transferred to a volumetric flask (250 mL capacity) and the final volume was completed with distilled water.

### 2.5. Preparation of the Total Polyphenols Solution

The stock solution above (2 mL) was placed into a volumetric flask (25 mL capacity) with 10 mL distilled water and 2 mL phosphomolybdic tungstic acid solution. The final volume was completed with a 29% Na_2_CO_3_ solution (w/v) and the absorbance was determined at 718 nm, after 30 min. The blank was prepared with all reagents, except the fluid extract stock solution.

### 2.6. Preparation of the Crospovidone (Insoluble Polyvinylpyrrolidone) Non-Adsorbed Polyphenols Solution

The stock solution (5 mL) was transferred to an Erlenmeyer (125 mL capacity) with 50 mg crospovidone, and the mixture was stirred for 60 min and filtered (minidisk). Then, 2 mL filtered solution were transferred to a volumetric flask (25 mL capacity) with 10 mL distilled water and 2 mL phosphomolybdic tungstic acid reagent, and the final volume was completed with 29% Na_2_CO_3_ (w/v). The absorbance was determined at 718 nm, 30 min after the addition of the Na_2_CO_3_ solution. For the blank, all reagents were used, except the stock solution.

## 3. Experimental Protocol

### 3.1. Animals

Male Wistar rats (250 g) from the Animal House of the Faculty of Medicine Estácio of Juazeiro do Norte (Ceará, Brazil) were divided into six groups (SO, untreated 6-OHDA and 6-OHDA treated with SEMU, at the doses of 5, 10, 20, and 40 mg/kg, p.o.), ranging from 5 to 12 animals. The treatments started 1 h after the stereotaxic surgery and continued daily for 14 days. The SO and untreated 6-OHDA groups were orally administered with distilled water, under the same experimental conditions as those of the 6-OHDA groups treated with SEMU. The animals were maintained in plastic cages, with food and water* ad libitum*, on a 12 h/12 h light/dark cycle, at a 23°C temperature. All experiments were carried out according to the Guide for the Care and Use of Laboratory Animals, USA, 2011. The project was approved by the Ethics Committee on Animals Experimentation of the Faculty of Medicine of the Federal University of Ceará, under the number 43/13.

### 3.2. Experimental Model of Parkinson's Disease (PD)

The classical method of intracerebral infusion 6-OHDA involves a massive destruction of nigrostriatal dopaminergic neurons and is largely used to investigate motor and biochemical dysfunctions in Parkinson's disease [11]. This unilateral rat model of PD resembles key features of human parkinsonian gait [12]. Thus, despite the availability of innovative models, the 6-OHDA model remains the most widely used for inducing a nigrostriatal lesion in the rat. This is due to its relatively low complexity and cost, the fact that the 6-OHDA-induced lesion is highly reproducible and the versatility of the procedure, yielding degrees of nigrostriatal lesions that develop with different temporal profiles, depending upon the site chosen for the neurotoxin injection [13]. In the present study, the animals were anesthetized with ketamine (80 mg/kg, i.p.) and xylazine (20 mg/kg, i.p.). They were then submitted to trichotomy of the superior portion of the head and fixed with bars into a stereotactic frame by their nose and ears. Next, a hole was made in the skull with a dentist drill, and the 6-OHDA (dissolved in saline, containing 0.2% ascorbic acid) was injected into the right striatum (two injections of 1 *μ*L, corresponding to a total of 12 *μ*g of 6-OHDA). The lesion was performed using a Hamilton syringe, at the following coordinates—AP: 0.9/1.4; ML: 3.8; DV: 3.3 mm from the bregma [14]. The injection was conducted at a rate of 0.5 *μ*L/min and the needle was left in place for 5 min, before it was slowly drawn back.

## 4. Behavioral Experiments 

### 4.1. Apomorphine-Induced Rotational Behavior

Currently, the reduction of apomorphine-induced rotational behavior in 6-OHDA-lesioned rats is the most utilized paradigm for assessing functional efficacy in this model of PD [15]. In the present work, the animals were submitted to the apomorphine-induced rotational behavior, fifteen days after the stereotactic surgery. One hour after the last drug administration, apomorphine (1 mg/kg, s.c.) was injected to the animals, and the number of contralateral rotations was scored for 60 min [16].

### 4.2. Rotarod Test

This test evaluates a potential motor incoordination caused by the drug, either by sedation or muscle relaxation [17]. Fifteen days after the stereotactic surgery and 1 h after the last drug administration (SEMU: 5, 10, 20, and 40 mg/kg, p.o.), each animal at a time was placed on a gyratory bar (12 rpm) and the number of falls/min was determined.

## 5. Neurochemical Experiments

### 5.1. Dopamine and DOPAC Determinations by HPLC

Parkinson's disease is characterized by the progressive loss of dopaminergic neurons, leading to decrease in striatal dopamine contents [18]. For the measurement of striatal monoamine levels, 10% homogenates were prepared in 0.1 M HClO_4_, sonicated for 30 s, and centrifuged at 4°C for 15 min at 15,000 rpm. Then, the supernatant was filtered (0.2 *μ*m, Millipore) and a 20 *μ*L sample was injected into a high-performance liquid chromatograph (HPLC) column (C18, 5 *μ*m, 250 × 4.6 mm). The mobile phase was 0.163 M citric acid, pH 3.0, containing 0.02 mM EDTA, with 0.69 mM sodium octanesulfonic acid (SOS), as ion pairing reagent, 4% v/v acetonitrile and 1.7% v/v tetrahydrofuran. DA and DOPAC were electrochemically detected, using an amperometric detector (Shimadzu, Japan), by oxidation on a glassy carbon electrode at 0.85V relative to the Ag-AgCl reference electrode. The concentrations of DA and DOPAC were determined by comparison with standards injected into the HPLC column at the day of experiment, and the results were expressed as ng/g tissue.

## 6. Histological and Immunohistochemical Assays

### 6.1. Cresyl Violet Staining

The method of Nissl uses the cresyl violet, a basic staining, to demonstrate neuronal cytoplasm, including the so called Nissl corpuscles. In the presence of neuronal lesions, these corpuscles can disappear, and then the staining serves as an indicator of neuronal viability, besides making the counting of remanescent neurons possible. Striatal sections were mounted in silanized slides and hydrated with xylol, followed by 100 down to 50% ethanol and distilled water (2 min). After that, the slides were immersed into the 0.5% cresyl violet solution and distilled water, and then by dehydration starting with 50% ethanol up to 100%. Finally, the slides were mounted beneath Entellan.

### 6.2. Immunohistochemistry for Tyrosine Hydroxylase (TH)

The reduction of TH expression is known to result in diminished dopamine synthesis leading to PD. Thus, this enzyme is essential in PD pathophysiology and its dysregulation will contribute to the disease aggravation [19]. Coronal slices of striata were used for TH immunohistochemical assays. After fixation in formaldehyde for 24 h, followed by 70% alcohol immersion, the slices were cut (5 *μ*m), placed on slides, and washed 3 times with PBS followed by the addition of 3% hydrogen peroxide in PBS. Then, the primary antibody anti-TH, from Chemicon, USA (1 : 200 dilution in 0.05 M Tris buffer, pH 7.2–7.6, containing 1% BSA), was added overnight. At the next day, the slices were washed 2 times with PBS, followed by the addition of the secondary antibody (yellow reagent or Link, DAKO Cytomation) for 1 h in a cold chamber. After that, the slides were washed again with PBS, followed by the addition of streptavidin-peroxidase (red reagent, DAKO Cytomation) for 40 min. After another wash, a DAB solution (3-3′-diaminobenzidine tetrahydrochloride, prepared according to the manufacturer's instructions) was applied on the top of the slices, for 30 s, and these were mounted on a free xylol medium.

### 6.3. Immunohistochemistry for GFAP (Glial Fibrillary Acidic Protein) and OX-42

The loss of dopaminergic neurons in PD and its consequent neuroinflammation is a chronic process, associated with a glial response composed of activated glial cells, including astrocytes and microglia [20, 21]. The immunoreactivity was detected by the avidin-biotin peroxidase technique. These immunohistochemistry assays were performed on striata slices (10 *μ*m) after fixation in formalin for 24 h, followed by immersion in 70% alcohol solution for slices processing, and the sections (5 *μ*m) were included in paraffin and placed in the oven at 60°C, for 3 h. After slices deparaffinization and hydration, the endogenous peroxidase blockade was carried out with H_2_O_2_. This step was followed by slices washing, for 5 min, with the 0.01 M buffer (from DAKO), and incubation with a rabbit polyclonal antisera against glial GFAP or a mouse monoclonal OX-42 antibody (both from Santa Cruz, USA), diluted according the manufacturers' instructions. Sections were then incubated for 2 h with either goat anti-rabbit or horse anti-mouse immunoglobulins (from Santa Cruz, USA), diluted 1 : 200, followed by incubation with the avidin-biotin peroxidase complex (diluted 1 : 100, Vectastin, Vector) for 90 min. Immunoreactivity was visualized by DAB (Sigma-Aldrich, USA), as the chromogen and H_2_O_2_ (0.05% v/v). The GFAP antibody recognizes the major protein of the cytoskeleton of astrocytes, and the OX-42 antisera identify the complement CR3 receptor and were used as a marker for microglia.

## 7. Statistical Analyses 

The results are expressed as means ± SEM. For multiple comparisons, the data were analyzed by one-way ANOVA, followed by Newman-Keuls as the* post hoc* test. The results were considered significant at *P* < 0.05.

## 8. Results

Table 1 presents the contents of tannins and polyphenols in the stem bark and stem and leaves of buds and shoots from the cultivated plant. Stem barks show the highest contents of all polyphenols. In general, buds (stem and leaves) present higher amounts of polyphenols, as related to shoots (also stem and leaves).

## 9. Behavioral Experiments

### 9.1. Apomorphine-Induced Rotational Behavior

**Figure 1:**
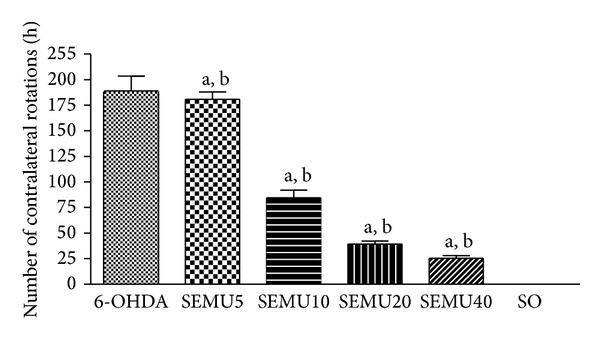
Effects of the standardized extract from* M. urundeuva* (SEMU) on the apomorphine-induced rotational behavior, in a rat model of Parkinson's disease. SEMU (5, 10, 20, and 40 mg/kg, p.o.) was administered, 1 h after the stereotaxic surgery (unilateral injection of 6-OHDA into the right striatum) and daily for 14 days. The animals were observed for 1 h, 30 min after the apomorphine injection (1 mg/kg, s.c.). The values are means ± SEM from 5 animals per group. (a) *P* < 0.01 versus 6-OHDA and (b) *P* < 0.001 versus SO (one-way ANOVA and Newman-Keuls as the* post hoc* test).

The 6-OHDA untreated group presented an average of 188 contralateral rotations, as related to none from the SO group. On the other hand, the 6-OHDA groups treated with SEMU (10, 20, or 40 mg/kg, p.o.) significantly decreased in a dose-dependent manner by 55, 79, and 87% the number of contralateral rotations/h, respectively, as related to the untreated 6-OHDA-lesioned group. No significant alteration was seen in the 6-OHDA group treated with the lowest dose of SEMU (5 mg/kg, p.o.), in relation to the untreated 6-OHDA group (Figure 1).

### 9.2. Rotarod Test

**Figure 2:**
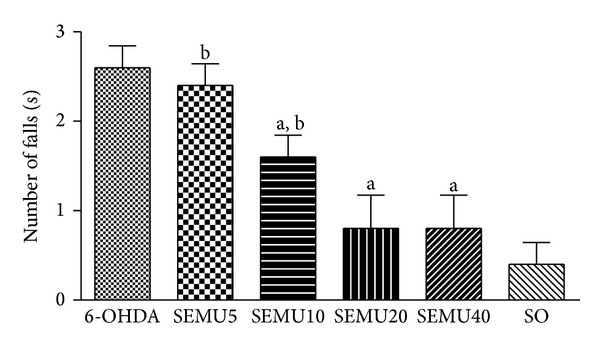
Effects of the standardized extract from* M. urundeuva* (SEMU) on the rotarod test, in a rat model of Parkinson's disease. SEMU (5, 10, 20, and 40 mg/kg, p.o.) was administered, 1 h after the stereotaxic surgery (unilateral injection of 6-OHDA into the right striatum) and daily for 14 days. The animals were placed upon a spinning bar (12 rpm), and the number of falls was determined for 1 min. The values are means ± SEM from 5 animals per group. (a) *P* < 0.01 versus 6-OHDA and (b) *P* < 0.001 versus SO, respectively (one-way ANOVA and Newman-Keuls as the* post hoc* test).

While the untreated 6-OHDA group showed an average of 2.6 falls/min, the 6-OHDA group treated with SEMU significantly decreased the number of falls by 39%, at the dose of 10 mg/kg, and percentages of reduction around 69% were seen with the two higher SEMU doses (20 and 40 mg/kg). Furthermore, the values with these doses were not different from that of the SO group. On the other hand, the untreated 6-OHDA group increased in 6.5 fold the number of falls, as related to the SO group, and similar results were observed with the 6-OHDA group treated with the lowest dose of SEMU (5 mg/kg) (Figure 2).

## 10. Neurochemical Experiments

### 10.1. DA and DOPAC Striatal Contents in the PD Model in Rats

**Figure 3:**
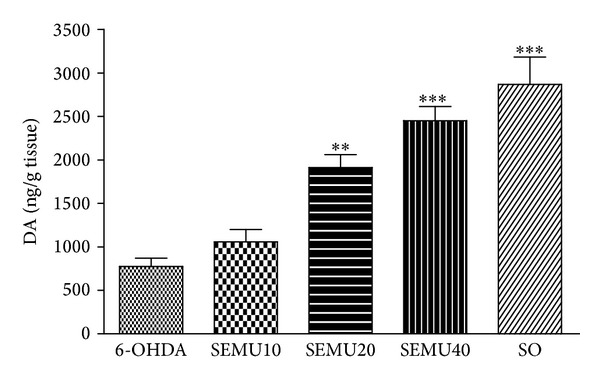
Effects of the standardized extract from* Myracrodruon urundeuva* (SEMU: 10, 20, and 40 mg/kg, p.o.) on dopamine (DA) levels in the right striatum. The drug was administered, 1 h after the stereotaxic surgery (unilateral injection of 6-OHDA into the right striatum) and daily for 14 days. The values are expressed as means ± SEM from 5 to 12 animals per group. *P < 0.05; **P < 0.01; ***P < 0.001 versus 6-OHDA (one-way ANOVA and the Newman-Keuls as the* post hoc* test).

The 6-OHDA lesion decreased by almost 70% DA contents in the ipsilateral side, as related to the unlesioned contralateral side, indicating an intense loss of dopamine neurons. On the other hand, a significant and dose-dependent recovery was demonstrated in the striatal levels of DA, in the 6-OHDA-lesioned group (ipsilateral side) after SEMU treatment, what was manifested mainly at the doses of 20 and 40 mg/kg. Interestingly, at the highest SEMU dose, no significant differences were observed among the DA contents in the ipsilateral sides of either 6-OHDA+SEMU40 or SO groups (Figure 3). While the DOPAC contents were not significantly altered in both sides of the SO group, they significantly decreased in the ipsilateral sides of the 6-OHDA groups treated with SEMU, at the doses of 20 and 40 mg/kg, as related to the ipsilateral side of the SO group. Surprisingly, while a significant increase was seen in the contralateral side of the 6-OHDA group treated with 40 mg/kg SEMU, relative to its ipsilateral side, the values of DOPAC were maintained lower in the 6-OHDA group treated with 20 mg/kg SEMU (Table 2).

### 10.2. DOPAC/DA Ratios

Significant increases in DOPAC/DA ratios were noticed in the untreated 6-OHDA group and the 6-OHDA group treated with SEMU (10 mg/kg), in their ipsilateral lesioned sides, as related to the unlesioned contralateral sides. While these ratios were somehow similar among both sides of the 6-OHDA groups treated with 10 (higher ratios) and 20 mg/kg (lower ratios), as related to the SO group, they were only lower in the ipsilateral side of the 6-OHDA treated with the highest SEMU dose (40 mg/kg) (Table 2).

### 10.3. Cresyl Violet Staining

**Figure 4:**
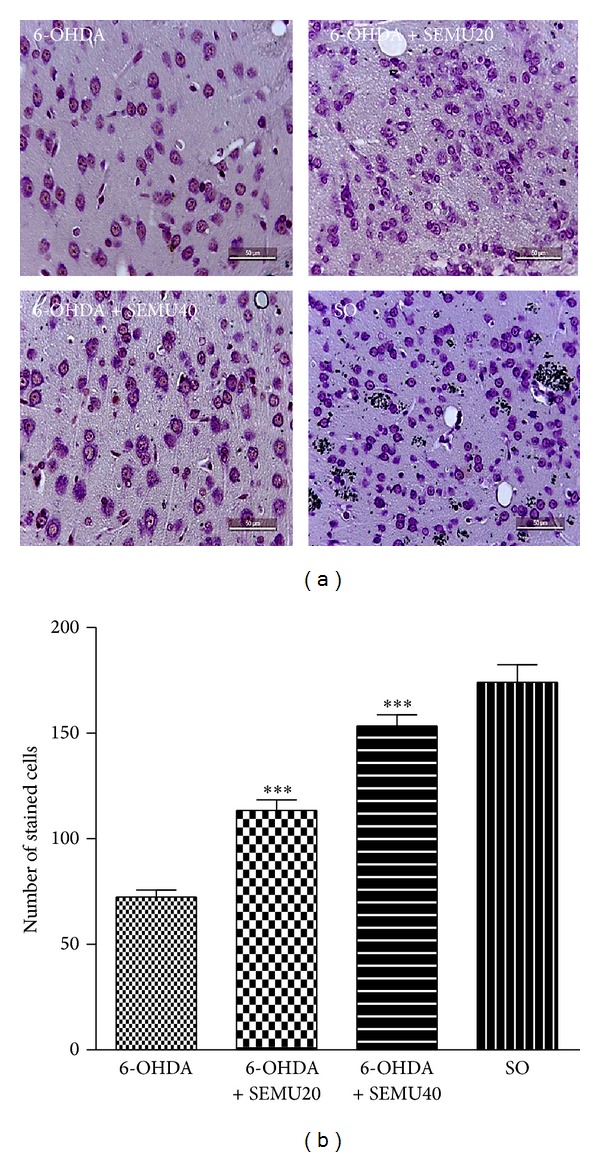
Photomicrographs of cresyl violet staining of the striatal tissue (lesioned right side) from untreated 6-OHDA and SEMU (20 and 40 mg/kg, p.o.) treated groups. The Sham-operated (SO) rats are the control group (a). A lower number of stained cells (indicating neuronal damage and nonviable neurons) are observed in the untreated 6-OHDA group, as related to the 6-OHDA groups treated with SEMU and mainly to the SO group (magnification: 400x). (b) shows the means ± SEM of the number of cells (three different fields), in each group. ***versus untreated 6-OHDA group (*F* = 59.85, *P* < 0.0001).

While lesser staining was demonstrated in the untreated 6-OHDA group, indicating a higher percentage of degenerated neurons, the reverse was seen in the 6-OHDA group after treatment with SEMU, at the doses of 20 and 40 mg/kg, and mainly in the SO group. These results indicate a better preservation of neurons and the presence of a greater number of viable cells, in the presence of SEMU treatments (Figure 4(a)).  Figure 4(b) shows that the number of cells increased 1.6 and 2.1 folds after SEMU treatments (20 and 40 mg/kg, resp.), as related to the untreated 6-OHDA group. The number of cells in the 6-OHDA treated with the higher SEMU dose was near to that of the SO group.

### 10.4. Immunohistochemistry for Tyrosine Hydroxylase

**Figure 5:**
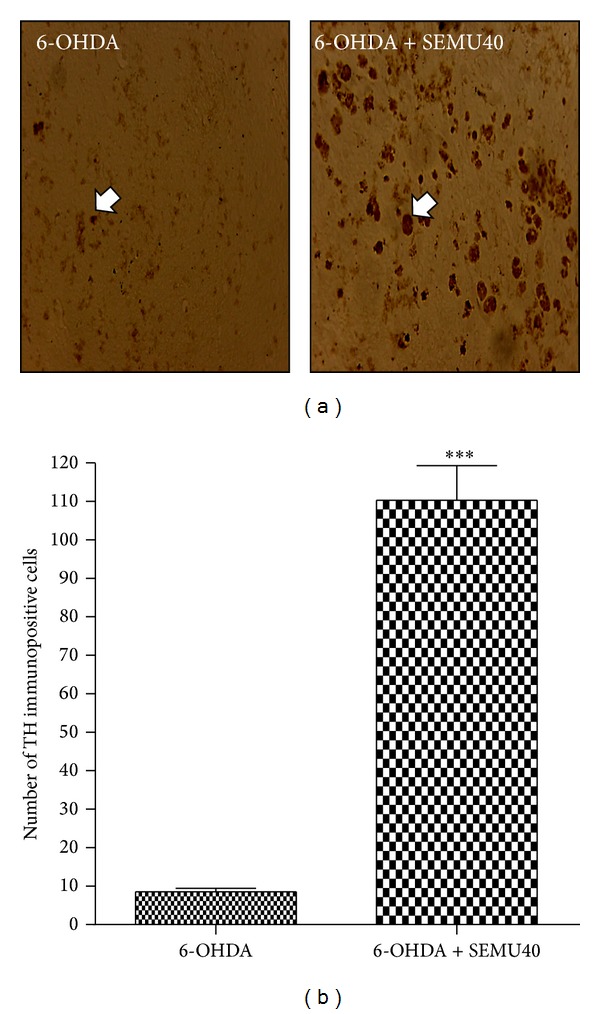
Immunohistochemistry for tyrosine hydroxylase (TH, a rate limiting enzyme for dopamine biosynthesis), in the lesioned right striatum (a). The untreated 6-OHDA group shows almost no TH immunopositive cells, as related to the 6-OHDA group after SEMU treatment, at the dose of 40 mg/kg, p.o. (magnification: 400x). Arrows indicate TH immunopositive cells. (b) shows the means ± SEM of the number of TH immunopositive cells (three different fields) in each group. ***versus the untreated 6-OHDA group (*t* = 16.74, df = 7.0, *P* < 0.0001).

The tyrosine hydroxylase enzyme (TH) is the rate-limiting step for dopamine synthesis. Its expression is greatly decreased in the presence of degeneration of dopaminergic neurons. Our results showed a low immunostaining for TH in the right striatum of the untreated 6-OHDA group, indicating the presence of a small number of immunopositive cells. This profile was altered in the striatum from 6-OHDA groups treated with SEMU, at the dose of 40 mg/kg, where a great number of TH positive cells were seen (Figure 5(a)).  Figure 5(b) shows the number of TH immunopositive cells, demonstrating an almost 13-fold increase in the 6-OHDA group after SEMU treatment, as related to the untreated 6-OHDA group.

### 10.5. Immunohistochemistry for GFAP and OX-42

**Figure 6:**
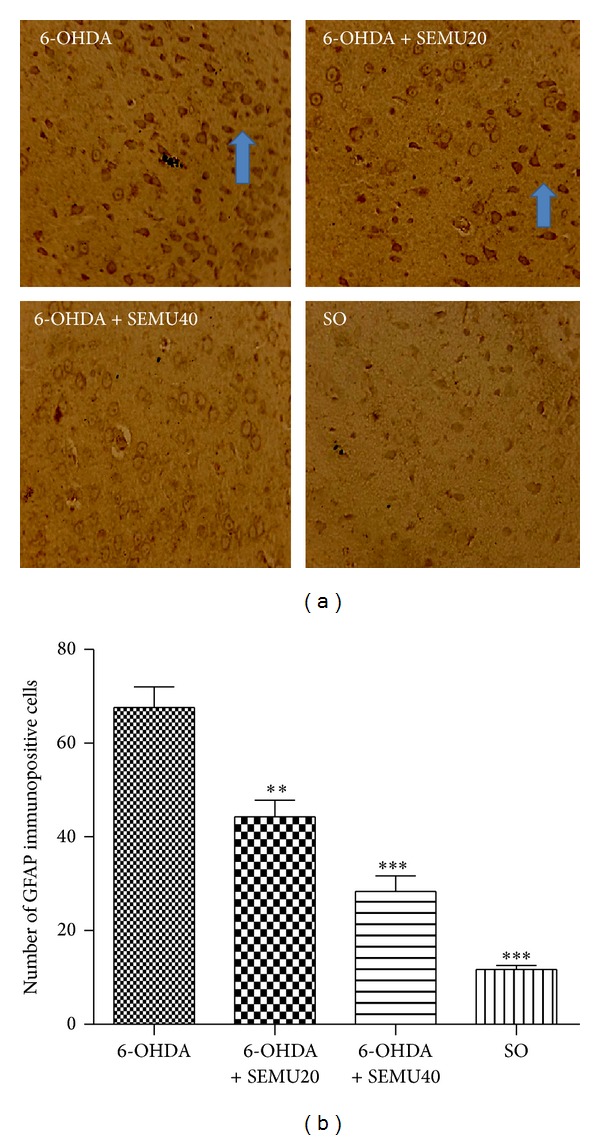
Immunohistochemistry for GFAP (a marker for astrocytes), in the lesioned right striatum (a). A high number of immunopositive cells are present in the untreated 6-OHDA group, and this number decreased in the 6-OHDA group after SEMU treatments (20 and 40 mg/kg, p.o.). Almost no immunopositive cells were noticed in the SO group (magnification: 400x). Arrows indicate GFAP immunopositive cells. (b) shows the means ± SEM of the number of GFAP immunopositive cells (three different fields) in each group. ***versus the untreated 6-OHDA group (*F* = 52.80, *P* < 0.0001).

**Figure 7:**
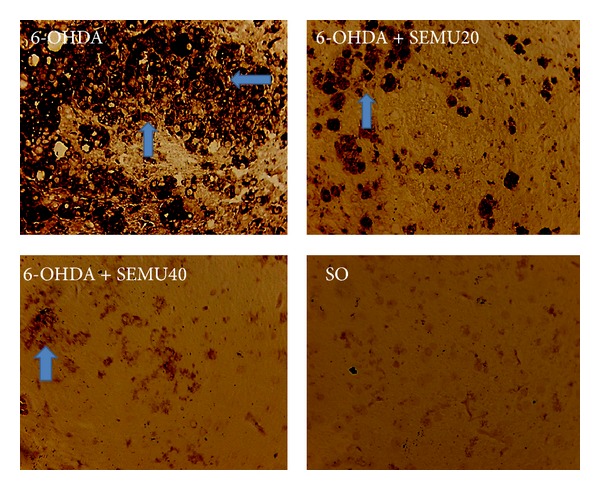
Immunohistochemistry for OX-42 (a marker for microglia), in the lesioned right striatum. A very high number of immunopositive cells are present in the untreated 6-OHDA group, as related to the 6-OHDA group after treatments with SEMU (20 and 40 mg/kg, p.o.). Almost no immunopositive cells were noticed in the SO group (magnification: 400x). Arrows indicate OX-42 immunopositive cells.

While almost no immunostained cells for either GFAP or OX-42 were shown in the SO group, increased numbers of astrocytes and microglia were observed in the lesioned right-striatum of the untreated 6-OHDA group. Figures 6(a) and 6(b) show the number of immunopositive cells for GFAP. The results demonstrated decreases of 35 and 58% in the number of GFAP immunopositive cells in the 6-OHDA groups, after treatments with SEMU at the doses of 20 and 40 mg/kg, respectively, as related to the untreated 6-OHDA group. A small percentage (12%) of GFAP immunopositive cells was detected in the SO group. A drastic increase in the number of OX-42 immunopositive cells was seen in the untreated 6-OHDA group. On the other hand, these cells were present in much lower numbers, in the 6-OHDA group after SEMU (20 and 40 mg/kg) treatments (Figure 7).

## 11. Discussion

In the present study, we showed for the first time the neuroprotective effects of a standardized extract from a Brazilian medicinal plant,* M. urundeuva*. This species stem bark is rich in chalcones, among other components. Chalcones belong to the flavonoid family, precursors of open chain flavonoids and isoflavonoids abundant in a variety of plant species. Chalcones and derivatives have attracted increasing attention, due to their numerous pharmacological activities, as antimalarial, anticancer, antiprotozoal, anti-inflammatory, antioxidant, and anticonvulsant actions, among others [22–24].

Previously, we showed that a chalcone-rich fraction isolated from* M. urundeuva* presents analgesic and anti-inflammatory activities, in experimental models of nociception and inflammation [4]. These effects were studied further with a tannin-rich fraction of this species, having anti-inflammatory and antiulcer properties [2]. Furthermore, chalcones from a plant stem bark fraction showed neuroprotective actions on 6-OHDA-induced neuronal cell death, in rat mesencephalic cells [25]. In the MTT assay, which is an index of cell viability, this fraction reversed the 6-OHDA-induced cell death and decreased lipoperoxidation and nitrite levels. Besides, the immunohistochemical analysis for tyrosine hydroxylase (TH) positive neurons indicated that 6-OHDA caused a concentration-dependent loss of TH+ and TH− neurons. On the other hand, the chalcone-rich fraction protected both cell types from 6-OHDA-induced cell death, demonstrating neuroprotective effects and reducing oxidative stress and apoptotic injury caused by 6-OHDA.

In the present study, these neuroprotective properties of* M. urundeuva* were shown, in the 6-OHDA experimental model of Parkinson's disease in rats. 6-OHDA was the first dopaminergic neurotoxin discovered, and also the most commonly employed in models* in vivo* and* in vitro. *It uses the same catecholamine transport system and induces neurotoxicity that is selective to these cells [26, 27]. However, the mechanisms responsible for the 6-OHDA-induced DA cell death have not been totally clarified, although the oxidative stress is clearly involved [28]. Thus, the formation of ROS by the autooxidation of 6-OHDA is considered as the main molecular mechanism underlying the neurotoxicity of 6-OHDA [29–31]. It has also been suggested that 6-OHDA acts as a direct inhibitor of the mitochondrial respiratory chain complex I [32, 33].

Behavioral tests in PD models can be used to characterize the extent of lesion or to detect therapeutic effects. Since the degree of neurotoxin-lesions is often variable, these tests can also serve to detect animals with an extend cell loss [34]. We showed a much lower number of apomorphine-induced contralateral rotations in the 6-OHDA group treated with SEMU, as related to the untreated 6-OHDA group. Apomorphine is a DA receptor agonist that, at low doses, causes contralateral turning by stimulating both supersensitive D1 and D2 receptors, preferentially in the denervated side. However, dopamine receptor upregulation does not occur, until a very high percentage of DA afferents is lost (80% or higher).

The SEMU treatment of the 6-OHDA groups decreased significantly the number of rat falls in the rotarod test. This is an established test used for the assessment of neurological deficits in rodents, following a pharmacological treatment. According to Iancu et al., 2005 [34], study with the unilateral 6-OHDA-lesion model of PD in mice, among the spontaneous motor tests, the rotarod test predicts nigral cell loss best. They showed that the time spent on the rotating bar correlated inversely with the cell loss, and these results agree with ours.

Furthermore, the drastic decrease of DA contents demonstrated in the lesioned right striatum of the untreated 6-OHDA group was reversed, in a dose-dependent manner, after SEMU treatments. A significant effect was already observed with the dose of 20 mg/kg, and DA levels similar to those of the SO group were seen in the 6-OHDA group treated with the highest dose of SEMU (40 mg/kg). Low levels of DOPAC, the main DA metabolite, were noticed in the ipsilateral side of the untreated and SEMU treated 6-OHDA groups, as related to the SO group.

In the present study, we found that DA levels in the contralateral striatum of lesioned rats were significantly higher than in the ipsilateral side, while DOPAC/DA ratios were lower in the contralateral striatum, suggesting a decreased DA turnover. Furthermore, DA levels in the ipsilateral striatum of lesioned rats were significantly lower and DOPAC/DA ratios higher, indicating an increased DA turnover. Increased striatal DOPAC/DA ratios have been already detected in the 6-OHDA model of PD [35]. These alterations in the ipsilateral striatum of the untreated lesioned rats were reversed after SEMU treatments. An increase in DA turnover has been assumed to occur early in Parkinson's disease, as a compensatory mechanism for dopaminergic neuronal loss [36]. Earlier studies [37] have shown that a relatively slower decrease in dopamine synthesis and a relatively faster increase in turnover in early disease probably act as compensatory mechanisms, and that the clinical onset of PD reflects a global failure of dopaminergic compensatory mechanisms. Although our results come from an experimental model, this compensatory mechanism could also be present.

Tyrosine hydroxylase is the rate-limiting enzyme responsible for converting tyrosine to L-Dopa, in the dopamine synthesis pathway. The pathophysiology of PD is largely due to dysregulation of the nigrostriatal dopaminergic system, with a decrease in TH activity, TH synthesis and TH mRNA, in the striatum of PD patients and in animal models as well [38]. This enzyme is therefore involved with the pathogenesis of PD, at several different levels, being a therapeutic target for the development of new disease treatments [18, 39]. We showed that while TH immunopositive cells were almost absent in the ipsilateral lesioned striatum, TH expression of the 6-OHDA group, after treatments with SEMU, was closer to that observed in the SO group. This indicated that the striatal injection of the 6-OHDA neurotoxin induces a massive disappearance of TH immunoreactive cells, and this effect was reversed by SEMU treatments, suggesting a neuroprotection against the 6-OHDA effect.

Furthermore, the number of cresyl violet staining cells was much lower in the 6-OHDA-lesioned right striatum, indicating a small number of viable neurons and a larger number of degenerated cells. On the other hand, a higher number of cresyl violet stained cells was demonstrated in the 6-OHDA groups, after SEMU treatments, and the results were similar to that seen in the SO group.

Microglia are resident immune-competent cells of the brain, and their expression and increased number led to the suggestion that immune mechanisms may play an important role in neurodegenerative diseases [40–42].* Postmortem* immunohistochemical analyses [43] performed for microglia activation showed an initially focal then widespread microglial response, in striatal and nigral levels, 4 weeks post-lesion. These authors used a model of Parkinson's disease induced by the intrastriatal administration of 6-OHDA, similar to ours, and concluded that inflammation is a significant component of progressive dopaminergic degeneration. Thus, drugs suppressing microglial activation are attracting attention as candidates for neuroprotection in PD [44].

In the present study, the observed drastic increases of OX-42 immunostaining, in the untreated 6-OHDA group, also indicate an inflammatory response in the lesioned striatum, and are consistent with an ongoing inflammatory response resulting from neuronal injury and neuronal death. The SEMU treatment of the 6-OHDA group reversed this neurotoxin effect and is most probably related to the anti-inflammatory action of SEMU, as detected by us (results not shown). We also observed similar results with the immunohistochemistry assays for GFAP. Not only OX-42, but also GFAP immunoreactive cells were demonstrated in the 6-OHDA model [45, 46]. These authors showed that the OX-42 immunoreactivity increased in the* substantia nigra*, 2 h after the 6-OHDA injection, peaked at day 7 and remained increased in the 14th day. The GFAP immunohistochemistry revealed that the increasing number and density of astroglial cells are dose-dependently related to 6-OHDA. Although, in the present case, we used constant doses of 6-OHDA, we observed a great number of GFAP immunopositive cells in the untreated 6-OHDA group, an effect also reversed after SEMU treatment.

Finally, for the first time, we showed a neuroprotective effect of SEMU treatments in a model of PD. Although, in the present study, we used a standardized extract of* M. urundeuva*, its main bioactive components are represented by tannins and chalcones, known for their antioxidant and anti-inflammatory properties. These effects were previously demonstrated by us in tannin- and chalcone-rich fractions [2, 4] and also in the standardized extract (results not shown). Furthermore, an* in vivo* and* in vitro* study [16] has shown protective effects from chalcone derivatives against neurodegenerative changes, by inhibiting oxidative stress and neuronal damage, what also occurred in the present study. Thus, the neuroprotection of the standardized extract from* M. urundeuva* could be the result of antioxidant and anti-inflammatory properties of its bioactive components, such as chalcones that, alone or in combination with current drugs, may represent an attractive strategy for treating or preventing neurodegenerative processes as those present in PD.

## Figures and Tables

**Table 1:** Percentages of total tannins, other polyphenols, and total polyphenol contents in cultivated *Myracrodruon urundeuva* Fr. All.

Plant part	Total tannins (%)	Other polyphenols (%)	Total polyphenols (%)
Stem bark	8.24	5.06	13.30
Bud stems	2.21	0.77	2.98
Shoot stems	0.99	0.76	1.75
Bud leaves	3.87	0.68	4.55
Shoot leaves	0.70	3.67	4.37

The values are expressed as gallic acid equivalents, determined in the fluid extract.

**Table 2:** Effects of the standardized extract from *M*.* urundeuva* (SEMU) on striatal DA and DOPAC levels (ng/g tissue), in the model of Parkinson's disease in rats.

Group	Dopamine (DA)	DOPAC	DOPAC/DA
6-OHDA ipsilateral	881.5 ± 108.77^a^	825.6 ± 121.2	0.94
6-OHDA contralateral	2559.0 ± 149.0	1141.0 ± 120.1	0.45
SEMU 10 ipsilateral	1057.0 ± 42.3	831.30 ± 239.8	0.79
SEMU 10 contralateral	2021.0 ± 37.57	1299.0 ± 167.1	0.64
SEMU 20 ipsilateral	1913.0 ± 146.2	208.2 ± 36.2^a,b^	0.11
SEMU 20 contralateral	2582.0 ± 280.2^b^	304.6 ± 46.6	0.12
SEMU 40 ipsilateral	2450.0 ± 1637^a,b^	314.7 ± 12.6^a^	0.13
SEMU 40 contralateral	3921.0 ± 221.8	1755.0 ± 172.0	0.45
SO ipsilateral	3186.0 ± 285.3^b^	1556.0 ± 227.7^b^	0.49
SO contralateral	2942.0 ± 204.0	1746.0 ± 196.6	0.59

The standardized extract from *M. urundeuva* (SEMU: 5, 10, 20, and 40 mg/kg, p.o.) was administered 1 h after the stereotaxic surgery (injection of 6-OHDA or distilled water into the right striatum-SO) and, afterwards, daily for 14 days. After behavioral experiments, all animals were euthanized for the dissection of right and left striata. The results are means ± SEM from 5 to 12 animals. ^a,b^ *P* < 0.05 versus the contralateral side of each group or versus the SO group (one-way ANOVA and the Newman-Keuls as the *post hoc* test).
